# Sequencing and characterization of the FVB/NJ mouse genome

**DOI:** 10.1186/gb-2012-13-8-r72

**Published:** 2012-08-23

**Authors:** Kim Wong, Suzannah Bumpstead, Louise Van Der Weyden, Laura G Reinholdt, Laurens G Wilming, David J Adams, Thomas M Keane

**Affiliations:** 1 Wellcome Trust Sanger Institute, Hinxton, Cambridge, CB10 1HH, UK; 2 The Jackson Laboratory, Bar Harbour, Maine, ME 04609, USA

## Abstract

**Background:**

The FVB/NJ mouse strain has its origins in a colony of outbred Swiss mice established in 1935 at the National Institutes of Health. Mice derived from this source were selectively bred for sensitivity to histamine diphosphate and the B strain of Friend leukemia virus. This led to the establishment of the FVB/N inbred strain, which was subsequently imported to the Jackson Laboratory and designated FVB/NJ. The FVB/NJ mouse has several distinct characteristics, such as large pronuclear morphology, vigorous reproductive performance, and consistently large litters that make it highly desirable for transgenic strain production and general purpose use.

**Results:**

Using next-generation sequencing technology, we have sequenced the genome of FVB/NJ to approximately 50-fold coverage, and have generated a comprehensive catalog of single nucleotide polymorphisms, small insertion/deletion polymorphisms, and structural variants, relative to the reference C57BL/6J genome. We have examined a previously identified quantitative trait locus for atherosclerosis susceptibility on chromosome 10 and identify several previously unknown candidate causal variants.

**Conclusion:**

The sequencing of the FVB/NJ genome and generation of this catalog has increased the number of known variant sites in FVB/NJ by a factor of four, and will help accelerate the identification of the precise molecular variants that are responsible for phenotypes observed in this widely used strain.

## Background

The origins of the FVB/NJ line can be traced back to an outbred colony of Swiss mice (N:GP, NIH general purpose) established at the National Institutes of Health (NIH) in the 1930s. A second colony (N:NIH) was subsequently established from the N:GP strain in the 1940s and then in the 1960s two new sub-lines (HSFS/N and HSFR/N) were established by selecting for sensitivity to histamine diphosphate challenge following pertussis vaccination. After eight generations of inbreeding, histamine-sensitive mice (HSFS/N) were found to carry the *Fv-l*^*b *^allele for sensitivity to the B strain of Friend leukemia virus [1,2]. These mice were bred to homozygosity and designated as the FVB/N inbred strain. FVB/N mice were imported to the Jackson Laboratories in 1988 at F37 and in 1991 were re-derived at N50 for addition to the Jackson foundation stocks. The FVB/NJ mouse has since become the workhorse of many mouse genetics laboratories due to its high reproductive performance, large litter sizes, and prominent pronuclei in fertilized eggs that make it an ideal strain for transgenic strain production [1,3]. FVB/NJ also represents an important disease model, and this strain has been used in a range of congenic and inter-strain crosses to identify disease-causing genes.

One well known phenotype of FVB/NJ mice is early onset retinal degeneration, which has been attributed to a nonsense mutation in the *Pde6b *gene [4]. FVB/NJ mice also fail to secrete complement 5 due to a 2-bp deletion in the *Hc *gene, which causes a truncation of the protein [1,5]. Resistance to collagen-induced arthritis, observed in FVB/NJ, has been shown to be due to a single nucleotide polymorphism, a 3-bp indel, and a large deletion in the T-cell receptor variable regions [6]. FVB/NJ mice also display a number of phenotypes relating to human disease, including susceptibility to seizures, diseases of the central nervous system [7], mammary hyperplasia [8] and with age, the spontaneous development of tumors in a range of organs, most commonly in the lung [9]. In addition, FVB/NJ mice are highly susceptible to chemically induced skin tumors [10], and are resistant to atherosclerosis [11].

Recent advances in sequencing technologies have reduced the cost of genome resequencing to a fraction of the cost required to produce the C57BL/6J mouse reference genome [12,13]. Next-generation sequencing technologies have been used to sequence the genomes of 17 common laboratory mouse strains, providing the most detailed picture of molecular variation between mouse strains to date [14,15]. This resource has already been used to accelerate the process of identifying candidate functional variants [16]. To complement this resource we sequenced the FVB/NJ genome. Here we describe a high quality catalogue of SNPs, insertion/deletion polymorphisms (indels) and structural variants (SVs) for this important strain. We compare these variants to those discovered in the 17 mouse genomes to profile the level of private variation in this strain, and demonstrate how our catalogue of FVB/NJ variants can be applied to identify and prioritize putative causative variants at quantitative trait loci (QTL) by examining the chromosome 10 atherosclerosis susceptibility locus, *Ath11*.

## Results and discussion

### SNPs and indels in the FVB/NJ genome

The FVB/NJ mouse genome was sequenced to a depth of approximately 50-fold coverage using 100-bp paired-end reads generated by the Illumina HiSeq 2000 sequencing platform [17] (European Nucleotide Archive accession ERP000687). The sequencing reads were mapped to the C57BL/6J mouse reference genome (NCBIM37/mm9) with BWA [18], followed by local realignment of reads around indels discovered by the Mouse Genome Project (MGP) using the Genome Analysis Toolkit (GATK) [19]. SNP and indel discovery was performed using the SAMtools mpileup function and BCFtools [20] (dbSNP handle: SC_MOUSE_GENOMES).

**Figure 1:**
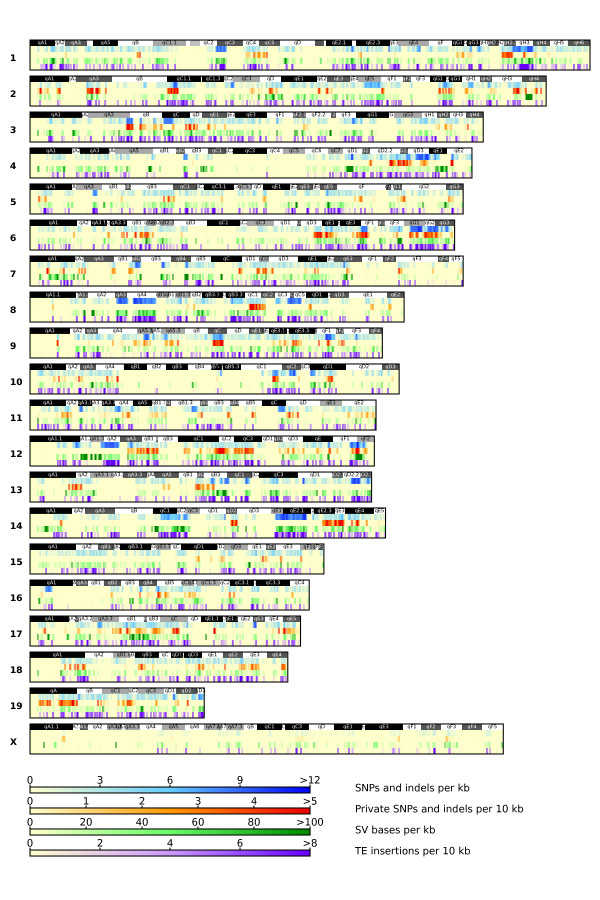
**Distribution of SNPs, indels and structural variants in the FVB/NJ genome, relative to the reference C57BL/6J genome**. Each rectangle represents a chromosome of the mouse genome. Shown in the top panel of each chromosome is the ideogram representation, followed by genome features, as indicated in the legend. SV, structural variant; TE, transposable element.

We have generated a catalogue of approximately 4.3 million high-confidence SNPs from the FVB/NJ sequence data (Figure 1). We randomly selected SNPs from this catalogue for genotyping using the Sequenom MassARRAY iPLEX Gold Assay [21] (Materials and methods), and estimate the false positive rate to be <1.6% (2/127; Materials and methods). We also compared our catalogue of SNPs to the list of FVB/NJ genotyped sites from the Perlegen/National Institute of Environmental Health Sciences (NIEHS) SNP set [22]. There are 996,981 homozygous non-reference SNPs for FVB/NJ in this data set, and of these, 91.7% (914,225) are present in our FVB/NJ catalogue. Of the 8.3% of SNPs that were not found in our FVB/NJ SNP catalogue, 2.7% were at sites classified as inaccessible (Materials and methods) and 5.6% were at accessible sites. To investigate this 5.6% discrepancy, we randomly selected sites from this list for genotyping using the iPLEX Gold Assay. The assays showed that a homozygous reference genotype was present at 96.8% (90/93) of the sites, suggesting that these are incorrectly genotyped as non-reference SNPs in the Perlegen/NIEHS data set. Taking this into consideration, we estimate that the majority of the 5.6% of SNPs in accessible regions that are not in our FVB/NJ catalogue are actually homozygous reference alleles, and therefore our false negative rate for accessible sites genotyped in the Perlegen/NIEHS study is <1%. It is important to note, however, that genome-wide the overall false negative rate will be higher as a large number of SNPs will fall in high copy repeats such as transposable elements and regions of low complexity [14].

We have also catalogued 0.82 million small indels in the FVB/NJ genome. The 0.38 million insertions range in size from 1 bp to 34 bp and 0.44 million small deletions range in size from 1 bp to 56 bp. We also used iPLEX Gold Assay to estimate the false positive rate of our indels. We tested a random sample of 100 short indels and estimate the false positive rate to be 10.1% (Materials and methods).

### Functional consequences of SNPs and indels

Using the Variant Effect Predictor from Ensembl [23], we assigned putative functional consequences to the variants found in FVB/NJ (Table 1). As expected, the majority of the SNPs do not fall inside of genes; just over half (2,247,487 SNPs, 52.1%) are classified as intergenic (>5 kb upstream or downstream of a gene), and 12.4% are within 5 kb of a protein-coding gene (345,321 SNPs) or non-coding gene (163,280 SNPs). Of particular interest are SNPs that affect splice sites and protein-coding regions, such as non-synonymous substitutions and those that generate or destroy stop codons. Based on Ensembl version 64 gene models, the number of SNPs causing either a premature stop codon or a stop codon loss was 71 and 16 SNPs, respectively. However, upon manual inspection of these sites, we were able to eliminate a number of these as true stop-gain and stop-loss variants due to incorrect gene models; these genes appear to be pseudogenes, non-coding genes, or artifacts (Tables S1 and S2 in Additional file 1). Exclusion of SNPs in these genes leaves 42 SNPs that generate a premature stop codon in all protein-coding transcripts of a gene. By manual inspection, we predicted whether the introduction of the premature stop codon would lead to either the production of a truncated protein (24 SNPs) or, if the premature stop codon is at least 50 bp upstream of a splice junction, nonsense-mediated decay (18 SNPs). Included in the list of true stop gain mutations is the previously identified C→A transversion at position 108,850,284 bp of chromosome 5 in the *Pde6b *gene. FVB/NJ is one of several mouse strains known to be homologous for this mutation, which has been shown to cause early onset retinal degeneration [4]. Ten SNPs appear to cause the loss of a stop codon in FVB/NJ (Table s2 in Additional file 1). Four of these are in polymorphic pseudogenes, where the mutation is actually a stop-gain in C57BL/6J. Manual inspection of the 19 sites affecting splice sites revealed that only ten of these actually affect the splice sites of valid protein-coding genes (Table s3 in Additional file 1); however, the effects of loss of the splice sites are difficult to predict.

**Table 1:** Predicted functional consequence of SNPs and indels

Consequence	SNPs	Indels
5 kb upstream or downstream	345,321	86,229
5' or 3' UTR	19,402	3,769
Intronic	864,790	173,237
Synonymous coding	13,697	NA
Non-synonymous coding	7,880	NA
Essential splice site	10^a ^(19)	0^a ^(3)
Stop gain	42^a ^(71)	NA
Stop lost	6^a ^(16)	NA
In-frame codon insertion or deletion	NA	140
Frameshift	NA	126
Two or more consequences	689,535	136,895
Within non-coding gene or mature microRNA	115,453	22,735
5 kb upstream or downstream of non-coding gene	163,280	31,988
Intergenic (>5 kb from a coding or non-coding gene)	2,247,487	416,289

A SNP or indel is annotated with a single consequence if the SNP or indel has the same effect on all protein-coding transcripts of a gene. A SNP or indel has two or more consequences if it has different effects on different transcripts of a gene. For example, a SNP may be in the intron of one transcript, and a synonymous SNP in a second transcript. Consequences were predicted using the Ensembl Variant Effect Predictor (VEP) [23] and gene models from Ensembl version 64. Note that a SNP may belong to more than one category. For example, a SNP may be a synonymous SNP in one gene and also within 5 kb of a second gene. ^a^The number of true stop codon gain, stop loss, and essential splice site consequences after manual inspection. The number in parentheses is the predicted number from VEP (Tables s1, s2 and s3 in Additional file 1). NA, not applicable.

There are 3,453 genes in which one or more non-synonymous SNPs affect all protein-coding transcripts (7,880 non-synonymous SNPs). To predict whether the amino acid substitutions would be deleterious, we assigned a Grantham matrix score (GMS) for each substitution. Only 5.2% of the non-synonymous substitutions are considered to be radical (GMS >150), and 11.8% are considered moderately radical (100 < GMS ≤ 150). We performed functional annotation of the 394 genes with radical substitutions using the Database for Annotation, Visualization and Integrated Discovery (DAVID) web-based tools [24,25], which showed a significant enrichment of genes associated with the PANTHER molecular function categories 'G-protein-coupled receptor' (*P <*1.5e-3), 'Receptor' (*P <*4.1e-3), and 'KRAB box transcription factor' (*P <*4.5e-3) (Table 2). The genes in these categories include several zinc finger proteins, olfactory receptors and vomeronasal receptors.

**Table 2:** Enrichment of PANTHER ontology terms from a list of 394 genes with radical amino acid substitutions (Grantham matrix score >150)

PANTHER term	Genes	Percentage of total	Fold enrichment	*P*-value
MF00002:G-protein coupled receptor	36	9.7	2.2	1.50E-03
MF00001:Receptor	50	13.4	1.7	4.10E-02
MF00224:KRAB box transcription factor	20	5.4	2.8	4.50E-03

Values were calculated using the Database for Annotation, Visualization and Integrated Discovery (DAVID) web-based tools [24,25]. The background gene list used to determine enrichment is all genes in the mouse genome. The *P*-value refers to the enrichment *P*-value, which is a modified Fisher's exact test. A Bonferroni multiple testing correction has been applied.

Similar to the SNPs, the majority of FVB/NJ indels are intergenic (50.4%) or within 5 kb upstream or downstream of a gene (14.3%) (Table 1). Only a small number of indels cause frameshifts (126) or in-frame codon losses or gains (140), including the known 'TA' deletion in the hemolytic complement gene (*Hc*; starting at position 34,898,728, chromosome 2), which is associated with susceptibility to allergen-induced bronchial hyper-responsiveness [5]. Manual inspection of the three indels predicted to affect splice sites revealed that two of the affected genes are likely to be a pseudogene and a long intergenic non-coding RNA, and the third is built from an incorrect gene model (Table s3 in Additional file 1).

### FVB/NJ private variants

We computationally determined the genotypes of the 17 MGP strains at all FVB/NJ variable SNP sites (Materials and methods) to identify SNPs and indels unique to FVB/NJ ('private' SNPs and indels). To obtain a high-confidence list, we classified a SNP site as private to FVB/NJ only if all 17 MGP strains were called as high quality homozygous reference alleles, as described for the FVB/NJ data in the Materials and methods. Using these criteria, we identified 115,228 private SNPs in FVB/NJ, which is 2.7% of the total number of SNPs called in this strain (Figure 1). From Sequenom genotyping of 103 sites, we estimate the false positive rate for private SNPs to be 3.0 to 5.8% (Materials and methods).

Nearly half of the private SNPs (48.9%) are found in intergenic regions, 21.2% are intronic, 8.6% are within 5 kb upstream or downstream of a coding gene, and nearly 17.1% are predicted to have 2 or more functional consequences. Among the remaining private FVB/NJ SNPs with a single consequence, there are 358 non-synonymous SNPs in 286 genes. Table 3 lists the private non-synonymous sites with a GMS of at least 150, excluding genes with unknown function (genes with Ensembl description 'predicted gene' and genes modeled from RIKEN cDNA). Included in the list, with a GMS of 205, is a non-synonymous substitution (C-to-A) in *Scn10a*, which changes a cysteine to a phenylalanine at residue 1,678. *Scn10a *is a voltage-gated sodium channel and mutations in this gene have been shown to produce several phenotypes [26]. The C-to-A substitution is located between two transmembrane regions at amino acid position 1,679 in an ion transport domain (Pfam domain PF00520), which makes this an interesting candidate for further functional studies. There are eight private SNPs that create a premature stop codon (Table s1 in Additional file 1), although manual inspection of the sites revealed that three of these generate truncated protein products (*Olfr228*, *Sh3gl3 *and *Ighv14-3*), three generate transcripts targeted by nonsense-mediated decay (*Bpifb9a*, *Gm5155*, *H2-Ab1*), while the remainder fall inside of genes with invalid Ensembl gene models. There are no variants private to FVB/NJ that cause the loss of a stop codon. Of the 10 SNPs affecting splice sites in the FVB/NJ genome, 3 are not found in the 17 MGP strains (*Spata21*, *Slc35a5*, *Gm4952*).

**Table 3:** Non-synonymous single nucleotide variants unique to FVB/NJ, relative to the 17 Mouse Genomes Project strains [14], with Grantham matrix score >150

Chromosome	Position	Ref/FVB	Gene ID	Gene name	Description	GMS
9	119518884	C/A	ENSMUSG00000034533	*Scn10a*	Sodium channel, voltage-gated, type X, alpha	205
4	138523178	G/T	ENSMUSG00000070661	*Rnf186*	Ring finger protein 186	205
19	13485488	G/T	ENSMUSG00000063777	*Olfr1469*	Olfactory receptor 1469	205
7	19600268	T/C	ENSMUSG00000040891	*Foxa3*	Forkhead box A3	194
15	101542878	C/A	ENSMUSG00000061527	*Krt5*	Keratin 5	184
9	85738244	C/T	ENSMUSG00000035274	*Tpbg*	Trophoblast glycoprotein	180
7	112715121	G/A	ENSMUSG00000037032	*Apbb1*	Amyloid beta (A4) precursor protein-binding, family B, member 1	180
6	43230658	C/T	ENSMUSG00000033542	*Arhgef5*	Rho guanine nucleotide exchange factor (GEF) 5	180
2	84642975	C/T	ENSMUSG00000027078	*Ube2l6*	Ubiquitin-conjugating enzyme E2L 6	180
19	3356468	C/T	ENSMUSG00000024900	*Cpt1a*	Carnitine palmitoyltransferase 1a, liver	180
19	16100043	C/T	ENSMUSG00000049247	*Rpl37-ps1*	Ribosomal protein 37, pseudogene 1	180
4	118859970	C/G	ENSMUSG00000028644	*Ermap*	Erythroblast membrane-associated protein	177
1	152466145	C/A	ENSMUSG00000066842	*Hmcn1*	Hemicentin 1	160
19	13137960	G/T	ENSMUSG00000060593	*Olfr1454*	Olfactory receptor 1454	159
19	12566519	C/A	ENSMUSG00000039982	*Dtx4*	Deltex 4 homolog (*Drosophila*)	159
18	14002581	C/A	ENSMUSG00000024420	*Zfp521*	Zinc finger protein 521	159
9	38623499	C/T	ENSMUSG00000043911	*Olfr922*	Olfactory receptor 922	155
1	168031022	T/A	ENSMUSG00000026564	*Dusp27*	Dual specificity phosphatase 27 (putative)	152

The C57BL/6J reference allele and FVB/NJ allele are shown (Ref/FVB). Predicted genes with unknown function are excluded. Gene IDs, names, and descriptions are from Ensembl version 64. GMS, Grantham matrix score.

We also identified 8,172 private indels using the criteria described above. Similar to the private SNPs, the majority of private indels are intergenic (45.6%), intronic (24.0%), within 5 kb of a coding gene (11.4%), or have multiple consequences (18.0%). Only seven of these affect all transcripts of a protein-coding gene: two are in-frame codon insertions or deletions and five result in frameshifts. All of the affected genes are olfactory receptors, with the exception of an in-frame 3-bp deletion in *Hr*, the hairless gene.

### Structural variants

There are few characterized structural variants in FVB/NJ in the literature. Two well-known examples include an approximately 6-kb deletion in *Bfsp2 *shared with 129 mice that results in alterations in lens optical quality [27], and a large genomic deletion resulting in the loss of segments of the T-cell receptor V beta, which has been shown to confer resistance to collagen-induced arthritis [6]. We have generated a catalogue of 30,048 structural variants (≥50 bp), which includes 15,358 deletions and 14,246 insertions, and combinations of SVs with complex paired-end mapping patterns (Figure 1 andTable 4) as described in Yalcin *et al. *[15,28]. To identify structural variants from basic paired-end mapping patterns and changes in mapped read depth, we used BreakDancer [29], CND [30], SECluster (unpublished) and RetroSeq [31] in the SVMerge [32] pipeline (Database of Genomic Variants archive accession estd200). Using the *de novo *assembly pipeline in SVMerge with reads surrounding breakpoints, we were able to refine the breakpoint coordinate predictions for 85% of the SVs. Similar to the genomes of the classical inbred strains sequenced by the MGP [14], structural variants affect less than 2% of the FVB/NJ genome. There are 328 SVs that overlap with protein-coding regions of 415 genes (149 of these genes are deleted or duplicated in their entirety); however, almost half (199) of these genes are annotated as 'predicted genes'. Functional annotation of the 415 genes using the DAVID web-based tools revealed a significant enrichment (*P <*0.01) of genes associated with the PANTHER gene ontology terms 'Defense/immunity protein,' 'Immunoglobulin' and 'Immunoglobulin receptor family member' after a Bonferroni correction was applied.

**Table 4:** Structural variants in the FVB/NJ genome

	Deletion	Duplication	Inversion	Insertion	Deletion in gain	Deletion + insertion	Inversion + deletion or insertion
Total	15,358	137	67	14,246	58	111	71
Genes	280	109	1	20	2	0	4

Listed are the number of SVs by type, and the number of genes with at least one protein-coding exon overlapping a SV. The following SV types involve more than one SV and have been described by Yalcin *et al. *[15,28]: a 'deletion in gain' is a deletion that is found within a duplicated region; a 'deletion plus insertion' is a deletion that co-occurs with an insertion; and an 'inversion plus a deletion or insertion' is an inversion flanked by one or more deletions or insertions.

When compared to the 17 strains sequenced in the MGP, 8,060 SVs in FVB/NJ were not identified in any other strain, with the majority (6196, 76.8%) of these being insertions. This list is likely to be composed of true private FVB/NJ insertions, true insertions in FVB/NJ that were missed in one or more of the 17 MGP strains, and false insertion calls. We used two software programs to call insertions: RetroSeq [31] and SECluster (unpublished) (Materials and methods). In order to estimate the false discovery rate of our insertion calls, we randomly selected 50 insertions from calls made by both RetroSeq and SECluster and 50 insertions each from calls made exclusively by RetroSeq and SECluster for PCR validation, giving 150 insertions in total. An insertion in the FVB/NJ genome was not observed for 6 of the 120 (5%) primer pairs that produced a band in both the reference C57BL/6J and FVB/NJ. From this we can infer that the majority of the approximately 8,000 insertions observed in FVB/NJ and not the 17 MGP strains are likely to be real. The false negative rates from the MGP showed that insertions were more difficult to identify than deletions, with false negative rates ranging from 24% to 32% for insertions, compared to 15% to 20% for deletions in 7 founder strains of the heterogeneous stock [15]. Therefore, a large portion of the approximately 8,000 insertions present only in FVB/NJ may be false negatives in one or more of the 17 MGP strains, rather than true private insertions. Several factors contribute to our improved sensitivity for identifying large insertions in FVB/NJ, compared to the MGP: higher sequencing depth (approximately 50× coverage compared to an average 25×), longer read length (100-bp HiSeq reads compared to primarily 54- to 76-bp Genome Analyzer II reads), and improvements in read alignment algorithms (BWA versus MAQ). Additionally, both RetroSeq and SECluster were also used in the MGP; however, both software have been adapted to take advantage of the additional information provided by both the longer reads and the ability of BWA to align portions of reads that flank insertion breakpoints (Materials and methods).

### Characterization of two narrowed regions in the Ath11 QTL

The search for genes of interest in QTL mapping experiments involving FVB/NJ mice is greatly facilitated by having a complete catalogue of genome-wide variants. To this end, we have re-examined a QTL for atherosclerosis susceptibility on chromosome 10, *Ath11*. QTLs for atherosclerosis susceptibility have been identified on chromosomes 1, 10, 14, 15 and 18 using inter-crosses between C57BL/6J (atherosclerosis-susceptible) and FVB/NJ (atherosclerosis-resistant) mice on *Apoe^-/- ^*and *Ldlr^-/- ^*knockout backgrounds [33-35]. Further studies of *Ath11 *using subcongenic mice allowed Wolfrum *et al. *[34] to refine the congenic interval (58.3 Mb) into two smaller regions, 10a (7.3 Mb), which is female-specific with 21 genes, and 10b (1.8 Mb), which contains 7 genes and is operative in both genders. The authors also examined differential expression of genes in these intervals using aortic tissues from F1 offspring. Wolfrum *et al. *searched for candidate causative SNPs in the two regions by mining public data resources (primarily dbSNP128) for sites at which C57BL/6J and FVB/NJ differed. They identified 22 SNPs affecting coding regions, intronic splice sites, or 5' or 3' UTRs. Additionally, they identified 31 potentially polymorphic sites, those which are known to be polymorphic but of unknown genotype in FVB/NJ. The authors listed genes of interest in the 10a and 10b regions as those on the Cardiovascular Gene Ontology Annotation Initiative's list of cardiovascular-associated genes: *Ipcrf1*, *Oprm1*, *Mtrf1l*, *Syne1*, *Esr1*, *Mthfd1l*, *Pde7b*, *Myb*, *Aldh8a1*, and *Sgk1*. The *Esr1 *gene, estrogren receptor α, in the 10a female-specific region was noted as a promising candidate gene, as it was identified as a regulator of one of the two gene networks identified from differentially expressed genes in the aortas of atherosclerosis-resistant and atherosclerosis-susceptible congenic mice. However, their database search revealed only one synonymous SNP and one potential synonymous SNP between FVB/NJ and C57BL/6J in *Esr1*. *Aldh8a1*, which was differentially expressed between congenic strains, was found to have one synonymous SNP and one 3' UTR SNP, but did not have any non-synonymous SNPs. In the 10b region, only one non-synonymous SNP was found in *Myb*, and another potential non-synonymous SNP was found in *Hbs1l*, while the remaining SNPs were synonymous SNPs or affected an intronic splice site or 5' or 3' UTRs.

**Figure 2:**
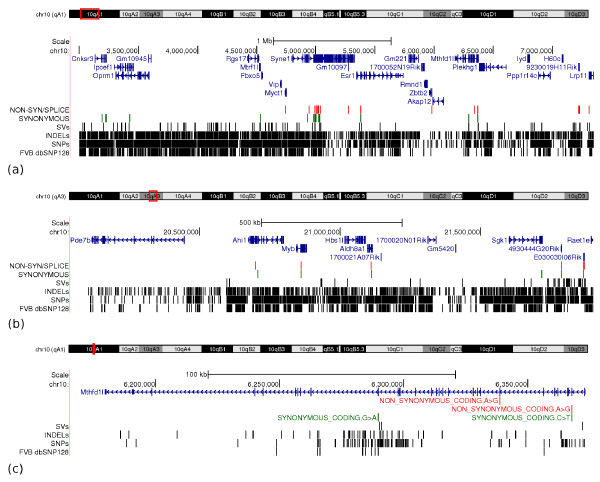
**SNPs, indels, and structural variants (SVs) in the FVB/NJ mouse, in two narrowed regions of the chromosome 10 *Ath11 *locus**. **(a) **The 10a locus from 1 to 7.3 Mb. **(b) **The 10b locus from 20.1 to 21.9 Mb. **(c) **Within the 10a region is the *Mthdf1l *gene, with non-synonymous and synonymous SNPs shown. Shown in red are non-synonymous SNPs or SNPs that affect intronic splice sites. In green are synonymous SNPs. In blue are gene models based on Ensembl version 64. Previously known FVB/NJ SNPs from dbSNP version 128 are also shown. The 10a region displayed in (a) begins at 3 Mb since the sequence from 1 bp to 3 Mb has not been assembled in the reference genome. Shown in the 10b region are the genes described in Wolfrum *et al. *[34] (*Pde7b*, *Ahi1*, *Myb*, *Hbs1l*, *Aldh8a1*, *Sgk1*), and additional predicted genes from Ensembl (*1700020N01Rik*, *1700021A07Rik*, *Gm5420*, *4930444G20Rik*, *E030030I06Rik*).

With the FVB/NJ genome sequence, we now have a comprehensive catalogue of SNPs for these regions, in addition to indels and structural variants (Figure 2). In the 10a and 10b regions, we have identified approximately 2,750 indels, 101 SVs, and 14,211 SNPs, which is approximately four times more SNPs than dbSNP128 contains for FVB/NJ in these regions. Of the known polymorphic sites in genes listed in Wolfrum *et al. *[34], only 2 out of 22 SNPs were not identified in our call set. Both of these SNPs had been filtered out of our call set due to their location in a region with high read depth (Materials and methods). We were also able to verify that only 8 of the 31 potentially polymorphic sites Wolfrum *et al. *identified do indeed differ between FVB/NJ and C57BL/6J (Table s4 in Additional file 1), significantly decreasing the number of candidate causative SNPs.

From our catalogue of variants, we have identified 12 additional non-synonymous SNPs in the 10a and 10b regions of the FVB/NJ genome (Table 5 and Figure 2). Eight of the 12 sites have previously been described as polymorphic in other mouse strains (dbSNP128), and all 12 SNPs are also present in at least one of the other 17 strains from the MGP. In the 10a region, we identified a single non-synonymous SNP in the ligand-binding domain of ENSMUSP00000101213, which is one of five protein products encoded by *Esr1*. We have also identified non-synonymous SNPs in three other genes, *Zbtb2*, *Lrp11 *and *Mthfd1l*, which had been omitted as genes of interest due to the lack of comprehensive SNP data. The two substitutions in *Mthfd1l *(Figure 2c) may be functional, as they fall inside of the binding domain and catalytic domain of this enzyme. This is an interesting gene candidate since SNPs in the human ortholog, *MTHFD1L*, have been associated with disease [36-38], including coronary artery disease [39-42]. No structural variants appear to affect protein-coding regions in 10a, although a total of 27 insertions and deletions fall in introns of 11 genes. In the 10b region, there is one non-synonymous SNP in *Aldh8a1 *that affects one of the two protein-coding transcripts. The SNP is located in the first base of the last exon in ENSMUST00000159163. This transcript, however, is based on a single expressed sequence tag (EST BI145926.1) and the coding sequence start and end have not been annotated. Non-synonymous SNPs are also present in the Ensembl predicted protein-coding genes *4930444G20Rik *and *E030030I06Rik*, and an indel also deletes four codons in the *4930444G20Rik *gene. Manual inspection of these genes, however, revealed that *E030030I06Rik *and *4930444G20Rik *are both pseudogenes. Here we have focused on variants in coding regions, although we also identified 467 variants in proximal promoter regions (within 5 kb upstream of a gene). Our catalogue of SNPs, indels, and SVs now provides the critical information for the functional analyses required to reveal the underlying factors responsible for atherosclerosis susceptibility in this QTL.

**Table 5:** Non-synonymous SNPs between C57BL6/J and FVB/NJ in *Ath11 *

Chromosome 10 position	Ref/FVB	dbSNP ID	MGP	Gene name, Ensembl gene ID and description	**Trans**.	Protein domain
5016217	G/C	None	8	*Syne1* ENSMUSG00000019769 Synaptic nuclear envelope 1	1/4	Spectrin repeat
5380387	C/A	rs29315913	13	*Esr1* ENSMUSG0000001976 Estrogen receptor 1	1/5	Nuclear hormone receptor, ligand-binding, core
5977100	A/C	None	16	*Zbtb2* ENSMUSG00000075327 Zinc finger and BTB domain containing 2	2/2	None
6338741	A/G	rs29378605	15	*Mthfd1l* ENSMUSG00000040675 Methylenetetrahydrofolate dehydrogenase (NAD P+ dependent) 1-like	3/3	Tetrahydrofolate dehydrogenase/cyclohydrolase, NAD(P)-binding domain
6367833	A/G	rs29320259	14	*Mthfd1l* ENSMUSG00000040675 Methylenetetrahydrofolate dehydrogenase (NAD P+ dependent) 1-like	3/3	Tetrahydrofolate dehydrogenase/cyclohydrolase, catalytic domain
7309964	T/C	rs29376214	6	*Lrp11* ENSMUSG00000019796 Low density lipoprotein receptor-related protein 11	4/6	**Polycystic Kidney Disease**(PKD) domain
21108744	A/G	rs49757404	12	*Aldh8a1* ENSMUSG00000037542 Aldehyde dehydrogenase 8 family	1/2	Aldehyde dehydrogenase domain
21787374	C/T	rs47037014	8	*4930444G20Rik* ENSMUSG00000069712 RIKEN cDNA 4930444G20 gene	1/1	None
21787451	A/C	rs50088182	9	*4930444G20Rik* ENSMUSG00000069712 RIKEN cDNA 4930444G20 gene	1/1	None
21864292	C/T	rs49453000	8	*E030030I06Rik* ENSMUSG00000055657 RIKEN cDNA E030030I06 gene	1/1	None
21868313	T/C	None	9	*E030030I06Rik* ENSMUSG00000055657 RIKEN cDNA E030030I06 gene	1/1	None
21868315	T/A	None	9	*E030030I06Rik* ENSMUSG00000055657 RIKEN cDNA E030030I06 gene	1/1	None

Wolfrum *et al. *[34] characterized two narrowed regions, 10a and 10b, within *Ath11 *containing 28 genes. This table describes additional non-synonymous SNPs discovered by sequencing of the FVB/NJ genome. Listed are the reference allele and FVB/NJ allele (Ref/FVB), the dbSNP ID (build 128) for sites previously identified as polymorphic in other mouse strains, and the number of strains in the Mouse Genomes Project (MGP) with the same allele as FVB/NJ. Also listed is the number of protein-coding transcripts (Trans.) affected and the protein domain involved. Gene and protein domain models were obtained from Ensembl version 64.

## Conclusions

We have sequenced the FVB/NJ mouse genome and catalogued SNPs, indels, and SVs. For SNPs alone, our study has increased the number of known variant sites by a factor of four for the FVB/NJ strain and therefore will serve as a valuable resource, as the FVB/NJ mouse strain is widely used for the generation of transgenic mice and in QTL mapping. We have shown how this resource can be used to characterize a QTL, accelerating the identification of candidate causal variants.

## Materials and methods

### DNA sequencing and read alignment

FVB/NJ DNA was obtained from the Jackson Laboratories (#1800; pedigree: 10-00964; generation: F95pF98) from a female FVB/NJ mouse. DNA (1 to 3 μg) was sheared to 100 to 1,000 bp using a Covaris E210 or LE220 (Covaris, Woburn, MA, USA) and size selected (350 to 450 bp) using magnetic beads (Ampure XP; Beckman Coulter). Sheared DNA was subjected to Illumina paired-end DNA library preparation and PCR-amplified for six cycles. Amplified libraries were sequenced using the HiSeq platform (Illumina) as paired-end 100 base reads according to the manufacturer's protocol. Each sequencing lane was genotype checked against the Perlegen SNP calls using the SAMtools programs BCFtools/glfTools [20]. A list of libraries and sequencing statistics is available in Table s5 in Additional file 1.

Sequencing reads from each lane were aligned to the C57BL/6J reference genome (NCBI build M37/mm9) using BWA version 0.5.9-r16 and the parameters '-q 15 -t 2'. To improve SNP and indel calling, the GATK [19] 'IndelRealigner' was used to realign reads near indels from the MGP [14]. The BAM files were then re-sorted and quality scores were recalibrated using GATK 'TableRecalibration'. Finally, SAMtools 'calmd' was used to recalculate MD/NM tags in the BAM files. All lanes from the same library were then merged into a single BAM file using Picard tools [43] and PCR duplicates were marked using Picard 'MarkDuplicates'. Finally, the library BAM files were merged into a single BAM containing all FVB/NJ sequencing reads.

### SNP and indel discovery, and prediction of consequences

SNPs and indels were identified using the SAMtools mpileup function, which finds putative variants and indels from alignments and assigns likelihoods, and BCFtools [20], which applies a prior and performs the variant calling. The following parameters were used: for SAMtools mpileup '-EDS -C50 -d 1000' and for BCFtools view '-p 0.99 -vcgN'.

Variants and indels were filtered using 'vcf-annotate' from the VCFtools package [44]. Filters and cutoff values are listed in Table s6 in Additional file 1. These filters are designed to identify inaccessible or uncallable sites and remove false SNP and indel calls due to alignment artifacts. Only homozygous SNPs and indels were retained.

To determine the effect of variants and indels on transcripts, we used the Ensembl Variant Effect Predictor tool version 2.2 [23] against mouse gene models from Ensembl version 64. Grantham scores were also generated to predict the impact of the amino acid substitutions.

SNPs and indels that are unique to FVB/NJ were identified by realigning the 17 MGP data using BWA, and performing recalibration and realignment around indels, as described above for the FVB/NJ data. The 17 MGP strains were then genotyped at all FVB/NJ SNP and indel sites using the SAMtools mpileup and BCFtools pipeline described above. To obtain a list of high confidence private FVB/NJ SNPs, we required each site to be genotyped as a homozygous reference allele in all 17 MGP strains, with a genotype quality of at least 30 and supporting read depth of 5 or more reads. The same criteria were applied to find private FVB/NJ indels.

### Sequenom validation

Genotyping was performed using the iPLEX™ Gold Assay (Sequenom^® ^Inc.) [21]. Assays for all SNPs were designed using the eXTEND suite and MassARRAY Assay Design software version 3.1 (Sequenom^® ^Inc.). Primers were designed from 100 bp of sequence flanking the SNP or indel of interest; FVB/NJ SNPs and indels in the flanking regions were masked, in addition to repetitive regions. Amplification was performed in a total volume of 5 μl containing approximately 10 ng genomic DNA, 100 nM of each PCR primer, 500 μM of each dNTP, 1.25× PCR buffer (Qiagen Crawley, West Sussex, UK), 1.625 mM MgCl_2 _and 1U HotStar Taq^® ^(Qiagen). Reactions were heated to 94°C for 15 minutes followed by 45 cycles at 94°C for 20 s, 56°C for 30 s and 72°C for 1 minute, then a final extension at 72°C for 3 minutes. Unincorporated dNTPs were SAP digested prior to iPLEX™ Gold allele specific extension with mass-modified ddNTPs using an iPLEX Gold reagent kit (Sequenom^® ^Inc.). SAP digestion and extension were performed according to the manufacturer's instructions with reaction extension primer concentrations adjusted to between 0.7 and 1.8 μM, dependent upon primer mass. Extension products were desalted and dispensed onto a SpectroCHIP using a MassARRAY Nanodispenser prior to MALDI-TOF analysis with a MassARRAY Analyzer Compact mass spectrometer. Genotypes were automatically assigned and manually confirmed using MassARRAY TyperAnalyzer software version 4.0 (Sequenom^® ^Inc.).

### Estimation of false positive and false negative rates

The false positive rate for SNP and indel discovery in FVB/NJ was estimated by randomly selecting 150 SNPs and 100 indels for genotyping in FVB/NJ and C57BL/6J using the Sequenom MassARRAY iPLEX Gold Assay [9], as described above. A genotype call was made for 128/150 SNPs and 69/100 indels. The concordance rate was 98.4% (126/128) for SNPs and 89.9% (62/69) for indels, giving false positive rates of 1.6% and 10.1%, respectively. However, one of the two discordant calls was due to a heterozygous call from the iPLEX Gold Assay. As we expect SNPs in inbred mice to be homozygous, this is likely to be an erroneous genotype call. We excluded this site to calculate the lower boundary of our SNP false positive rate, 0.8% (1/127 discordant calls). We also selected 111 private FVB/NJ SNPs, of which 103 genotype calls were made. The private SNPs chosen for genotyping only included non-synonymous SNPs, SNPs that create premature stop codons, or SNPs affecting splice sites. The concordance rate was 94.2% (97/103). Three of the discordant calls were heterozygous calls by the iPLEX Gold Assay, and excluding these calls gives a concordance rate of 97% (97/100).

We compared our catalogue of FVB/NJ SNP calls to FVB/NJ genotypes from the Perlegen/NIEHS data set [22]. There are 996,981 sites genotyped as homozygous non-reference SNPs in this data set, and of these, 91.7% (914,225) are present in our FVB/NJ catalogue. Of the remaining, 2.7% (26,869) fell into 'uncallable' sites (see Materials and methods above) and 5.6% (55,887) were not in our SNP catalogue. We randomly selected 100 SNP sites from the 5.6% discordant sites for SNP genotyping using the iPLEX Gold Assay. Of the 93 that produced genotype calls, 90 sites (96.8%) were genotyped as homozygous reference alleles. Assuming that this portion of the 5.6% discordant genotypes are homozygous reference bases, then our false negative rate at accessible sites in the Perlegen/NIEHS data set is less than 1%. Two of the three discordant genotypes were called by the iPLEX Gold Assay as heterozygous SNPs in the FVB/NJ sample.

### Structural variant discovery

SVs were identified using SVMerge [32], in which we applied a combination of BreakDancer [29], CND [30], RetroSeq [31] and SECluster (unpublished), followed by filtering and local sequence assembly for all deletions and insertions (from SECluster) to obtain exact breakpoints, all as described previously [15,28]. For this analysis, SECluster was optimized to work with reads mapped with BWA [18] by including mate pairs with one mate soft-clipped and read pairs with non-inward facing orientation for read clustering and insertion calling. RetroSeq was also updated to include soft-clipped reads in the breakpoint resolution step. The parameters for all SV callers are shown in Table s7 in Additional file 1. Tandem duplications, inversions, and more complex paired-end mapping patterns were also identified using in-house Perl scripts, as described in Yalcin *et al. *[28]. Mouse genome reference assembly gaps, centromere and telomere regions were obtained from the University of California Santa Cruz Table Browser [45]. Structural variants overlapping these regions, plus a window on either side of 500 bp for assembly gaps and 20 kb for centromeres and telomeres, were excluded, as mapping artifacts in these regions cause false SV calls.

### DAVID analysis

For analysis of genes with radical amino acid substitutions, a list of 394 Ensembl gene IDs was submitted to the DAVID website [24,25] (version 6.7) for functional annotation, and 368 mapped to genes in their database. For structural variants overlapping genes, a list of 415 Ensembl gene IDs was submitted to the DAVID website, and 331 mapped to genes in their database. The default EASE threshold score was used (0.1) and the minimum number of genes for a term was set to 2.

### Primer design and PCR analysis

PCR primers to validate insertions were designed using Primer3 release 2.2.3 [46] and an in-house Perl script. The optimal primer length was set at 20 bp and all other Primer3 defaults were used. FVB/NJ SNP sites and structural variants were masked. Primers were designed to have a product size between 200 and 1,000 bp, relative to the reference genome. Candidate primer pairs were checked for uniqueness in the mouse reference genome, and insertions with no unique primer pairs were excluded from validation.

PCR was performed on C57BL/6J and FVB/NJ genomic DNA (The Jackson Laboratory, Bar Harbor, Maine, USA) using either Thermo-Start *Taq *DNA Polymerase (Abgene, Epsom, UK) with an annealing temperature of 60°C and extension time of 30 s (for 35 cycles) or Platinum^® ^*Taq *DNA Polymerase High Fidelity (Life Technologies Limited, Paisley, UK) with an annealing temperature of 60°C and extension time of 5 to 8 minutes (for 35 cycles), according to the manufacturers instructions. The PCR products were run on 1 to 2% agarose gels containing ethidium bromide, and visualized using a UV transilluminator. The approximate sizes of the PCR products were calculated by running molecular weight markers (Hyperladder™ I; Bioline Reagents Limited, London, UK) on each gel.

### Comparison of SNPs in at the *Ath11 *locus

We used the BioMart tool from Ensembl build 64 to retrieve genotypes for the FVB/NJ strain in the chromosome 10 *Ath11 *10a and 10b regions, from positions 1 to 7,300,000 bp and 20,100,000 to 21,900,000 bp. We queried the Ensembl *Mus musculus *Variation 64 database, which is generated from dbSNP Build 128 [47]. From this list we identified sites with non-reference alleles, and compared these variants to the set of FVB/NJ variants we generated as described above.

## Abbreviations

bp: base pair; DAVID: Database for Annotation: Visualisation and Integrated Discovery; GATK: Genome Analysis Toolkit; GMS: Grantham matrix score; MPG: Mouse Genome Project; NIEHS: National Institute of Environmental Health Sciences; NIH: National Institutes of Health; PCR: polymerase chain reaction; QTL: quantitative trait locus; SNP: single nucleotide polymorphism; SV: structural variant; UTR: untranslated region.

## Competing interests

The authors declare that they have no competing interests.

## Authors' contributions

KW, TMK and DJA conceived the ideas for the study. KW carried out discovery and analysis of variants and SVs, ran comparative analyses, and interpreted results. TMK carried out the quality control, processing and mapping of the sequencing data, interpreted results, and ran insertion calling using RetroSeq. SB performed genotyping assays for SNP and indel sites. LVDW performed PCR experiments for insertion validation. LW performed the manual inspection of variants and gene models in Ensembl. LR provided essential biological materials. KW, DJA, and TMK wrote the manuscript, which was approved by all of the authors.

## Supplementary Material

Additional file 1 **Supplemental tables**. Click here for file
