# Supplementary material for: Sequencing and characterization of the FVB/NJ mouse genome
Source: Genome Biol. 2012 Aug 23;13(8):R72. doi: 10.1186/gb-2012-13-8-r72 (PMC3491372; doi:10.1186/gb-2012-13-8-r72)
Supplement: Additional file 1 — Supplemental tables. [file gb-2012-13-8-r72-S1.PDF]

Supplemental Tables

Supplemental Table S1: Single nucleotide polymorphisms (SNPs) in FVB/NJ predicted to cause premature stop codons. SNPs that are >50bp upstream of the 3'-most exon splice site are likely to be targeted by nonsense mediated decay (NMD; highlighted in *green*), otherwise produce a truncated protein (highlighted in *yellow*). Upon inspection of the SNPs, however, we identified a number of genes are actually pseudogenes or non-coding (*orange* and *red*). In *magenta* are genes that have a stop gain but may re-initiate translation at a downstream ATG. Eighteen SNPs result in NMD and 24 result in truncated proteins. Positions marked with an asterisk (*) are SNPs which are not found in FVB/NJ but not in any of the 17 Mouse Genomes Project strains. Gene models and description are based on Ensembl release 64.

Chr	Position	Ref/Alt	Ensembl ID	Gene	Description
2	154088143*	G/T	ENSMUSG00000067998	*Bpifb9a*	NMD
4	136959762	C/T	ENSMUSG00000078520	*Gm13011*	NMD
5	108850284	C/A	ENSMUSG00000029491	*Pde6b*	NMD
6	41992170	G/T	ENSMUSG00000023289	*Sva*	NMD
6	123736831	G/A	ENSMUSG00000072780	*Vmn2r24*	NMD
6	124173938	C/T	ENSMUSG00000072778	*Vmn2r27*	NMD
7	18488075*	C/T	ENSMUSG00000078793	*Gm5155*	NMD
11	67177997	C/T	ENSMUSG00000060180	*Myh13*	NMD
12	115505913	G/A	ENSMUSG00000076671	*Gm16931*	NMD. 98->69 aa, but Ig variable gene segment so mature transcript
14	54501839	G/A	ENSMUSG00000076864	*Trdv1*	NMD. 112->95 aa but Ig variable gene segment so mature transcript
17	20492780	T/A	ENSMUSG00000056910	*Vmn2r107*	NMD
17	34401803*	C/G	ENSMUSG00000073421	*H2-Ab1*	NMD
17	36265874	G/C	ENSMUSG00000072377	*Gm7030*	NMD (same gene as H2-T9/ ENSMUSG00000092243)
19	7069213	A/T	ENSMUSG00000037349	*Nudt22*	NMD
1	141450194	G/T	ENSMUSG00000057037	*Cfhr1*	NMD
12	105504783	G/T	ENSMUSG00000079014	*Serpina3i*	NMD
17	36167998	C/A	ENSMUSG00000067212	*H2-T23*	NMD
2	86323290*	G/T	ENSMUSG00000075180	*Olfr228*	Single-exon gene so not NMD. Truncates to 2/3 of normal size.
2	86323121	A/T	ENSMUSG00000075180	*Olfr228*	313->203 aa
2	87890594	G/T	ENSMUSG00000075139	*Olfr1162*	314->62 aa
4	138429303	G/T	ENSMUSG00000078518	*Gm13030*	143->68 aa
4	143747782	C/A	ENSMUSG00000050810	*Oog3*	500->496 aa
5	13796418	G/T	ENSMUSG00000067855	*Speer3*	261->251 aa
6	42740724	C/T	ENSMUSG00000057108	*Olfr452*	317->229 aa
6	124141650	A/T	ENSMUSG00000072778	*Vmn2r27*	857->846 aa
6	141920824	C/A	ENSMUSG00000079263	*Gm6614*	650->616 aa or 670->616 aa
7	11333761	A/T	ENSMUSG00000059206	*Vmn1r71*	307->116
7	11748007	G/A	ENSMUSG00000090714	*Zscan4d*	SNP in last exon, truncates C-terminus by 244aa. Not NMD.

7	89455315*	C/A	ENSMUSG00000030638	*Sh3gl3*	SNP in last exon, truncates C-terminus by 62aa which includes the SH3 domain. Not NMD. Also, one variant EST BU841959.1 has different reading frame in last exon and SNP would be synonymous CGT>AGT.
8	19242065	C/A	ENSMUSG00000071169	*Defb46*	57->32 aa
8	124881040	G/A	ENSMUSG00000039079	*Trhr2*	382->374
10	21787507	G/A	ENSMUSG00000069712	*4930444G20Rik*	495->127 aa (retrogene)
11	58303250	C/A	ENSMUSG00000050813	*Olfr332*	344->336 aa
12	115298470*	C/T	ENSMUSG00000076660	*Ighv14-3*	Stop after 4aa from start so no NMD; alternative downstream ATG 6aa from stop.
14	52331633	C/A	ENSMUSG00000068506	*Gm5800*	186->177 aa
15	77465990	G/A	ENSMUSG00000091694	*Apol11b*	337->107 aa (one variant possibly escapes: it skips penultimate exon which results not in NMD, but a potential downstream CDS starting from in-frame ATG in last exon, after the SNP)
17	18462998	C/T	ENSMUSG00000079698	*Vmn2r93*	857->723 aa
17	21027477	G/A	ENSMUSG00000050933	*Vmn1r231*	311->47 aa
2	118588361	T/A	ENSMUSG00000045838	*A430105I19Rik*	SNP about 27bp downstream of CDS start; next available ATG in same exon, shortens peptide 545->500 aa
4	136965668	G/A	ENSMUSG00000078520	*Gm13011*	SNP 7bp downstream of CDS start; use of next downstream ATG shortens peptide 283->145 aa
7	92304338	G/T	ENSMUSG00000091901	*Vmn2r67*	SNP 74bp downstream of CDS start; use of next downstream ATG shortens peptide 851->765 aa
9	37854854	G/T	ENSMUSG00000058130	*Olfr884*	SNP 45bp downstream of ATG; use of next downstream ATG shortens peptide 38 aa
2	180067679	C/T	ENSMUSG00000056061	*Gm14318*	Doubtful whether protein-coding: would be NMD; Antisense to Gata5
4	155214164	T/A	ENSMUSG00000073679	*Gm10562*	Probably not coding: peptide doesn't look like anything else. Antisense to Mxra8
4	155214202	C/T	ENSMUSG00000073679	*Gm10562*	Probably not coding: peptide doesn't look like anything else. Antisense to Mxra8
6	48539964	C/A	ENSMUSG00000052730	*Gm5111*	Doubtful whether coding, but if coding 190->38 aa
10	93761114	C/T	ENSMUSG00000053420	*Gm4792*	Non-coding in EG, but could be coding; if coding: NMD
17	34669866	C/A	ENSMUSG00000061728	*Btnl7*	Considered pseudo in EG (no locus specific transcription), but if coding: 583->570 aa
1	89693078	G/T	ENSMUSG00000073623	*5830472F04Rik*	Not coding: artifact
1	173433735	T/A	ENSMUSG00000091993	*B930036N10Rik*	Not coding: antisense to Alyref2
2	93151714	C/T	ENSMUSG00000023461	*Tspan18*	Not a separate protein coding gene: overlaps, and is variant of, 5' UTR of Tspan18 ENSMUSG00000027217
3	148848278	A/T	ENSMUSG00000053583	*Gm9912*	Not coding: artifact
3	148887660	C/T	ENSMUSG00000070342	*Gm10287*	Not coding: lincRNA

5	104884532	A/T	ENSMUSG00000058736	*Gm10047*	Not coding: artifact
6	136434654*	G/A	ENSMUSG00000091408	*Gm6728*	Ensembl/refseq model invalid: processed pseudogene.
8	94870731	A/T	ENSMUSG00000031736	*4933436C20Rik*	Not coding, lincRNA
8	109643343	C/A	ENSMUSG00000040399	*C630050I24Rik*	Not coding, possibly lincRNA
9	41389469	T/G	ENSMUSG00000074415	*2610203C20Rik*	Not coding: lincRNA
9	58402971	G/A	ENSMUSG00000074273	*Gm10657*	Not coding: antisense to Cd276
9	115346933*	C/T	ENSMUSG00000074034	*Gm5921*	Processed pseudogene.
10	105629475	G/A	ENSMUSG00000056877	*Gm7263*	Processed pseudogene
11	6368695	A/T	ENSMUSG00000049647	*Purb*	Not coding: long 3' UTR of *Purb* or separate lincRNA
11	116655829	C/T	ENSMUSG00000091913	*Gm17558*	Not a real gene
12	75062354	C/T	ENSMUSG00000066457	*Gm5068*	S100a11 processed pseudogene (intact CDS but no locus-specific transcription)
12	81372647	A/T	ENSMUSG00000079585	*Gm17425*	Ensembl/RefSeq model invalid: artifact (single exon mRNA with 3' flanking genomic As). Translation invalid.
13	69682569	C/A	ENSMUSG00000051805	*A530095I07Rik*	Not coding: antisense to Nsun2
14	48847114	G/T	ENSMUSG00000063885	*Gm6498*	Transcribed GAPDH processed pseudogene
15	59826369	C/T	ENSMUSG00000071750	*Gm7713*	GAPDH processed pseudogene
15	82609924	G/A	ENSMUSG00000058613	*Gm5062*	Unprocessed cytochrome P450 pseudogene
16	87549630	G/A	ENSMUSG00000090753	*Rpl31-ps4*	Pseudogene
19	38127422	G/A	ENSMUSG00000069554	*I830134H01Rik*	Not coding: lincRNA

Supplemental Table S2: Single nucleotide polymorphisms (SNPs) in the FVB/NJ genome predicted to cause the loss of a stop codon. Six SNPs destroy a stop codon. Four SNPs are located in a polymorphic pseudogene, and are actually stop codon gains in C57BL/6J. In *red* are stop losses in genes which, upon manual inspection, are pseudogenes or are invalid gene models. Gene models and description are based on Ensembl release 64.

Chr	Position	Ref/Alt	Ensembl Gene ID	Gene	Description
5	109271968	A/C	ENSMUSG00000091624	*Vmn2r9*	Stop loss adds 16aa.
5	130112413	A/G	ENSMUSG00000029436	*Mmp17*	Stop loss adds 9aa.
6	122035856	T/C	ENSMUSG00000030131	*Mug2*	Stop loss adds 10aa.
9	22005066	T/C	ENSMUSG00000074472	*Zfp872*	Stop loss adds 90aa. Extra sequence adds more zinc fingers.
13	91936742	C/A	ENSMUSG00000021621	*Zcchc9*	Stop loss adds 2aa. Human ortholog agrees with B6.
19	42144825	T/A	ENSMUSG00000025176	*Hoga1*	Stop loss adds 1aa.
1	176082455	A/G	ENSMUSG00000091950	*Olfr421*	Polymorphic pseudogene. Stop loss adds 47aa. This is actually a stop gain SNP in B6 because locus-specific transcript has the FVB sequence (i.e. no stop), which yields the more canonical length peptide.
5	105755500	T/C	ENSMUSG00000092021	*Gbp11*	Polymorphic pseudogene. Stop loss adds 177aa. This is actually stop gain SNP in B6 (rs33735324) because stop locus specific evidence is like FVB; the loss restores translation to full-length.
7	108703363	A/G	ENSMUSG00000092517	*Art2a-ps*	Polymorphic pseudogene. Stop loss adds 127aa. This actually a stop gain SNP in B6.
7	110935829	A/G	ENSMUSG00000047535	*Olfr67*	Stop loss adds 6aa. Strain 129 and Celera seem same as FVB so possibly actually a stop gain SNP in B6.
1	21275691	A/G	ENSMUSG00000025936	*Gm4956*	Glutathione S-transferase, alpha (Gsta) family pseudogene (transcribed unprocessed). Neither of two transcript variants yields a viable protein. Some parent sequence missing. Stop codon just after parent ATG; Ensembl model uses next in-fame ATG just after stop, but also shifts a splice acceptor upstream (exon 4). Another, more obvious, Gsta pseudogene just upstream.
5	53000987	T/C	ENSMUSG00000052295	*8030423F21Rik*	Novel transcript (lincRNA). Ensembl CDS not believable: 106aa CDS, mostly LTR repeat, in last of 3 exons.
7	111881958	A/G	ENSMUSG00000051885	*Olfr664*	Pseudogene. Designated pseudogene because of loss of canonical ATG and 19aa premature stop. No locus specific transcripts.
8	94870703	A/G	ENSMUSG00000031736	*4933436C20Rik*	Novel transcript (lincRNA). Ensembl CDS not believable: 106aa, would be NMD.
9	106783843	T/C	ENSMUSG00000074102	*Rbm15b*	Not actually a separate gene, let alone coding: part of extended 3' UTR of the real Rbm15b
12	81372647	A/T	ENSMUSG00000091579	*Gm17373*	Not a real gene: single cDNA with 3' genomic As, so artefact. Ensembl translation tiny 50aa and low complexity.

Supplemental Table S3: Single nucleotide polymprphisms (SNPs) and indels affecting splice sites. Listed below are SNPs and indels in the FVB/NJ genome located in the first two or last 2 bases of an intron. Only SNPs which affect all splice variants are shown. Reference genome and FVB/NJ alleles are shown (Ref/Alt). SNPs found in the FVB/NJ genome and not the 17 Mouse Genomes Project strains are indicated by an asterisk (*). Sites listed in grey boxes are found, after manual inspection, to be in non-coding genes, pseudogenes, or genes built from invalid gene models.

Chr	Position	Type	Ref/Alt	Ensembl ID	Gene	Comment
2	49786505	SNP	G/A	ENSMUSG00000026765	*Lypd6b*	Possibly leads to retained intron but probably doesn't affect CDS
3	36378490	SNP	G/A	ENSMUSG00000027713	*1810062G17Rik*	Possibly leads to retained intron and if it finds another donor to NMD. If doesn't find another donor, it doesn't lead directly to NMD but would have very large 3' UTR as this is the last intron.
4	140667182*	SNP	G/A	ENSMUSG00000045004	*Spata21*	If it skips that exon it would yield NMD transcript.
6	117820273	SNP	G/A	ENSMUSG00000042097	*Zfp239*	Doesn't affect CDS: the alternative coding variant isn't valid as it had an unsupported CDS totally different from the Znf CDS. One or two variants not affected.
9	51126490	SNP	T/C	ENSMUSG00000036027	*1810046K07Rik*	If it skips that exon doesn't affect CDS. One variant with different 5' UTR exon may not be affected (can't tell for sure as variant may not be complete)
10	93353840	SNP	A/T	ENSMUSG00000036112	*Metap2*	Only affects a minority variants: one variant that has alternative 5' UTR extra exon that has TSS here but at a much lower prevalence than the downstream TSS with unspliced 5' UTR; one variant that has two extra 5' UTR exons where the affected exon is 2nd and where the TSS is least common; and one variant where the completeness of the model can't yet be determined but as it is annotated now the affected exon is the first.
11	114660332	SNP	G/T	ENSMUSG00000034677	*Gpr142*	Possibly leads to retained intron/NMD transcript.
16	45157760*	SNP	C/G	ENSMUSG00000022664	*Slc35a5*	Indeed a coding exon for a minority variant, 5' UTR exon for majority of variants. For the latter the SNP does not directly affect the CDS but in all likelihood the disabling of this splice has disastrous consequences for the CDS. Note that two splice variants, that have an alternative donor more upstream and more downstream respectively, are not affected.
19	11239541	SNP	C/T	ENSMUSG00000024728	*1700025F22Rik*	One variant unaffected because it skips this exon; another variant with downstream first exon probably also unaffected but we don't know how complete that variant is so can't tell for sure.

19	12701202*	SNP	A/G	ENSMUSG00000071633	*Gm4952*	Last coding exon: effect difficult to predict.
13	115399214	SNP	G/A	ENSMUSG00000051758	*4930544M13Rik*	Gene is almost certainly a lincRNA: the CDS in the Ensembl model is questionable as it is small, doesn't look like anything else, exons aren't conserved and one exon overlaps a repeat. Moreover, that CDS (or start of that CDS) doesn't work in another splice variant.
19	61193631	SNP	C/T	ENSMUSG00000074732	*Zfp950*	Not actually a coding gene despite its name. Only affects 2 variants: two other variants skip this exon and 3 retained intron variants don't contain this sequence.
6	129419013	SNP	A/G	ENSMUSG00000079293	*Clec7a*	Actually a STOP gain in BL6 (or STOP loss in FVB); Ensembl model incorrect as it moved this acceptor and exon 2 donor to circumvent the stop codon. No locus specific mRNAs, but two apparently locus specific ESTs that skip this exon, which yields a valid translation as the exon is in-frame. So this is a polymorphic pseudogene with a coding variant.
7	79818826	SNP	T/C	ENSMUSG00000070035	*Gm4885*	Actually a processed pseudogene. The Ensembl model is not correct, based on incorrect alignment of a splice variant of the parent that circumvents the single in-frame stop codon. The gene is very conserved (99.7% nt, 100% aa)
10	77454832	SNP	G/C	ENSMUSG00000020277	*Pfkl*	This was a sequence error which isn't yet corrected in Ensembl/VEGA but is correct in the HAVANA assembly. The “SNP” in FVB actually represents the correct sequence with correct CAG splice site (in stead of CAC).
10	93757926	SNP	A/G	ENSMUSG00000053420	*Gm4792*	Changes splice donor from G-GT to G-GC so remains splice site. Note that this gene is also affected by a stop SNP in exon 1.
10	94306815	SNP	G/C	ENSMUSG00000074785	*Plxnc1*	This was a sequence error which isn't yet corrected in Ensembl/VEGA but is correct in the HAVANA assembly (by moving the assembly switchpoint so that this sequence now comes from the BAC with the right sequence). The “SNP” in FVB actually represents the correct sequence with correct A-GT splice site (in stead of A-CT).
14	52191232	SNP	T/C	ENSMUSG00000091142	*Gm17175*	Restores splice site (G-AT > G-GT), i.e. BL6 has the non-splice site: almost certainly a sequence error in BL6 as SNP rs48025647 doesn't have any frequency data and all other strains in Mouse Genomes have the canonical splice.
17	24539981	SNP	G/T	ENSMUSG00000073439	*Gm10506*	Actually not a gene: entirely contained within a SINE repeat

7	46519724	Indel	TCCTTACCTGAC/ TC	ENSMUSG00000074169	*Gm16387*	ENSMUSG00000074169 is possibly a (polymorphic?) pseudogene. The best matching BL6 mRNA AK030037 and even PCR-rescued BC150918 have quite a few mismatches with reference. If it is a real gene, the splice donor of exon 1 would be destroyed, probably giving rise to a retained intron-NMD transcript. Note that there are many (pseudogene) copies of this gene upstream and downstream.
8	94870818	Indel	CT/C	ENSMUSG00000031736	*4933436C20Rik*	lincRNA; destroys splice acceptor; some variants unaffected
9	40792439	Indel	CTTGATCTGAA/ C	ENSMUSG00000032021	*Crtam*	Not actually a splice SNP: the Ensembl model and the CCDS model have been incorrectly built because of alignment problems with non BL6 mRNAs like AF001104.1These non-BL6 transcripts (though they don't state which strain they come from) also have quite a few SNPs on other exons.

Supplemental Table S4: Genotypes of 8 of the 31 sites previously described as potentially polymorphic by Wolfrum *et al*. [1], in the *Ath11* 10a and 10b regions. Genotype information for FVB/NJ was previously not available at the 31 sites. Listed below are the site which we have identified as non-reference bases in FVB/NJ. The reference genome and FVB/NJ alleles are shown (Ref/FVB), the number of strains from the Mouse Genomes Project (MGP) [2] with the same allele as FVB/NJ, the functional consequence of the SNP and the number of transcripts with the specified consequence. All gene and protein annotations were obtained from Ensembl build 64.

Chr 10 position	Ref/FVB	MGP	Consequence	Gene	No. of transcripts affected	Affected Protein Domain
5007247	G/A	6	Non-synonymous	*Syne1*	2/4	Spectrin repeat superfamily
5019159	T/G	6	Non-synonymous	*Syne1*	1/4	Spectrin repeat superfamily
5274496	G/T	15	Non-synonymous	*Syne1*	1/4	Spectrin repeat superfamily
5377292	T/A	10	Synonymous	*Esr1*	1/5	Ligand-binding domain of nuclear hormone receptor
7221350	G/A	16	Non-synonymous	*9230019H11Rik*	1/1	MHC antigen-recognition domain superfamily
7221512	C/T	13	Non-synonymous	*9230019H11Rik*	1/1	MHC antigen-recognition domain superfamily
7226334	T/C	16	Non-synonymous	*9230019H11Rik*	1/1	None
21891803	G/A	5	Intronic	*H60b/Raet1e**	1/2	None

*Ensembl build 64 gene models differ than RefSeq

Supplemental Table S5: FVB/NJ sequencing libraries and sequencing statistics. GB is gigabases.

Library	Mean Insert Size (bp)	Cycles	Mapped bases (GB)
FVB_offspring2_200a 2686245	173	104	4.8
FVB_offspring2_200b 2686246	166	104	4.7
FVB_offspring2_200b 3411721	167	79	26.2
FVB_offspring2_200c 2686247	171	104	5.8
FVB_offspring2_200d 2686248	188	104	5.8
FVB_offspring2_200d 3411722	185	79	27.0
FVB_offspring2_200e 2686249	190	104	4.5
FVB_offspring2_200f 2686250	255	104	12.2
FVB_offspring2_200f 3411723	251	79	32.0
MWGS 3293661	337	104	75.4

Supplemental Table S6: Values for filters used on FVB/NJ raw variant and indels calls.

Filter Tag	Description	Info Tag(s)	Value
StrandBias	Minimum *p*-value for strand bias	PV4	0.0001
EndDistBias	Minimum *p*-value for end distance bias	PV4	0.0001
MinDP	Minimum read depth	DP or DP4	5
MaxDP	Maximum read depth	DP or DP4	199
BaseQualBias	Minimum *p*-value for baseQ bias	PV4	0
MinMQ	Minimum RMS mapping quality for SNPs	MQ	25
Qual	Minimum value of QUAL field	QUAL	10
MinAB	Minimum alternate bases	DP4	2
VBD	Minimum Variant Distance Bias	VDB	0
GapWin	Window size for filtering adjacent gaps	PV4	3
MapQualBias	Minimum *p*-value for mapQ bias	PV4	0
SnpGap	SNP within INT bp around a gap to be filtered	-	10
RefN	Reference base is N	-	-
HWE	Minimum *p*-value for HWE (plus F<0)	HWE and G3	0.0001

Supplemental Table S7: Parameters used in structural variant calling.

Software	Version	Parameters	Additional Filters
BreakDancerMax	1.1 (25/10/2010)	bam2cfg.pl: -c 3 -n 50000<br>BreakDancer: -c 3 -m 10000000 -q 25	Score: 25<br>Read support: 2<br>Copynum (deletions): 2
CND	1.1	CND: --smooth 100 --repeat-cutoff=0.35	None
RetroSeq	1.1	Defaults	Filter hits within 50bp of a RepeatMasker elements of same type

REFERENCES:

Wolfrum S, Rodriguez JM, Tan M, Chen KY, Teupser D, Breslow JL: **The mouse atherosclerosis locus at chromosome 10 (*Ath11*) acts early in lesion formation with subcongenic strains delineating 2 narrowed regions.** *Arteriosclerosis, thrombosis, and vascular biology* 2010, **30:**1583-1590.

Keane TM, Goodstadt L, Danecek P, White MA, Wong K, Yalcin B, Heger A, Agam A, Slater G, Goodson M, et al: **Mouse genomic variation and its effect on phenotypes and gene regulation.** *Nature* 2011, **477:**289-294.
